# Effects of acute aerobic exercise on circulating sTLR and sRAGE profiles in normal‐ and abnormal‐glucose‐tolerant individuals

**DOI:** 10.14814/phy2.15859

**Published:** 2023-11-20

**Authors:** Ryan K. Perkins, Edwin R. Miranda, Pallavi Varshney, Sarah S. Farabi, Lauretta T. Quinn, Jacob M. Haus

**Affiliations:** ^1^ Department of Kinesiology California State University, Chico Chico California USA; ^2^ School of Kinesiology University of Michigan Ann Arbor Michigan USA; ^3^ Center for Human Nutrition Washington University School of Medicine St. Louis Missouri USA; ^4^ Goldfarb School of Nursing at Barnes‐Jewish College St. Louis Missouri USA; ^5^ Department of Behavioral Health Sciences University of Illinois at Chicago Chicago Illinois USA

**Keywords:** diabetes, exercise, inflammation, metabolic disease, sRAGE, sTLR

## Abstract

BMI‐matched normal‐ (NGT, *n* = 10, 41 ± 4y, 35.6 ± 3.0 kg/m^2^) and abnormal‐glucose‐tolerant (AGT, *n* = 16, 51 ± 3y, 34.3 ± 1.5 kg/m^2^) participants were evaluated for body composition, metabolic health (oral glucose tolerance test [OGTT]), and VO_2_max. Participants also completed a treadmill walking test at 65% VO_2_max for 30 min. Total sRAGE, esRAGE, sTLR2, and sTLR4 were assessed via ELISA, and cRAGE was calculated. AGT exhibited greater (*p* < 0.05) body fat % (+24%), fasting plasma glucose (+37%), OGTT AUC (+59%), and HOMA‐IR (+55%) and lower (*p* < 0.05) VO_2_max (−24%). sTLR2 was 33% lower in AGT than NGT (main effect, *p* = 0.034). However, sTLR2 did not change (*p* > 0.05) following AE. sTLR4 tended to be 36% lower in AGT than NGT (main effect, *p* = 0.096) and did not change following AE (*p* > 0.05). Total sRAGE and isoforms were similar (*p* > 0.05) between groups and did not change following AE (*p* > 0.05). sTLR2 was correlated with (*p* < 0.05) basal BG (*r* = −0.505) and OGTT AUC (*r* = −0.687). sTLR4 was correlated with basal BG (*p* < 0.10, *r* = −0.374) and OGTT AUC (*p* < 0.05, *r* = −0.402). Linear regressions were predictive of sTLRs in the basal state (sTLR2: *R* ^2^ = 0.641, *p* = 0.01; sTLR4: *R* ^2^ = 0.566, *p* = 0.037) and after acute exercise state (sTLR2: *R* ^2^ = 0.681, *p* = 0.004, sTLR4: *R* ^2^ = 0.568, *p* = 0.036).These findings show circulating sTLR profiles are disrupted in AGT and acute AE minimally modulates their levels.

## 1 INTRODUCTION

Impaired glucose tolerance manifesting as pre or overt diabetes is a growing public health concern (Magliano et al., 2019). Not only is the estimated 37 million individuals in the United States with diabetes at risk of reduced quality life (Prevention CfDCa, 2023), but this metabolic disorder is suspected to lead to development of other diseases (Kilhovd et al., 2007; Koska et al., 2018; Shah & Brownlee, 2016) thereby exerting a profound economic burden. In fact, care for individuals with diabetes was recently estimated to account for 1 in every 4 health care dollars in the United States and over half of that is directly attributed to diabetes (Association, 2017). A primary consequence of the development and progression of insulin resistance is the perpetuation of inflammatory signaling through several well‐studied pathways, including the receptor for advanced glycation end products (RAGE) and Toll‐like receptor (TLR) isoforms (Cassese et al., 2008; Creely et al., 2007; Giacco & Brownlee, 2010; Jialal et al., 2014; Schmidt et al., 2001).

RAGE is a pattern recognition receptor (PRR) that through its action promotes oxidative stress and inflammation. Upon ligand binding via advanced glycation end products (AGEs) and other pathogen‐ (PAMP) and damage‐associated molecular patterns (DAMPs), RAGE activates the nuclear factor‐κB (NFκB) and other pathways (Nielsen et al., 2017; Schmidt et al., 1994, 2001; Tanaka et al., 2000). Interestingly, RAGE‐NFκB signaling functions as a positive feedback mechanism as the NFκB pathway promotes transcription of RAGE, thus perpetuating a futile inflammatory cycle.

Much like RAGE, TLRs are also PRRs. Ten TLRs (TLR1‐10) are expressed in human skeletal muscle (Nishimuru & Naito, 2005; Perkins et al., 1985; Pillon & Krook, 2017). Of the 10 TLRs that exist in humans, many function in line with RAGE as a result of recognizing similar PAMPs/DAMPs, convergence on the intracellular adaptor protein (i.e., Myd88), and activation of the NFκB pathway (Ducharme et al., 2022; Federico et al., 2020; Kawai & Akira, 2010; Nielsen et al., 2017). More specifically, TLR2 and TLR4 are two of the most highly expressed TLRs in human skeletal muscle (Perkins et al., 1985; Pillon & Krook, 2017) and each has been shown to recognize PAMPs/DAMPs that are commonly shown to be elevated in insulin‐resistant individuals, such as lipoproteins, high mobility group box 1 (HMGB1), lipopolysaccharide (LPS), and AGEs (Choi et al., 2012; Tsan & Goa, 2004). In fact, RAGE and TLR4 are suspected to heterodimerize upon activation, highlighting their interdependence and shared downstream pathway mechanisms (Nielsen et al., 2017).

RAGE, TLR2, and TLR4 signaling can be disrupted via proteolytic cleavage of the extracellular or ectodomain by matrix metalloproteinases (MMPs) thereby appearing in the cell media in culture or the circulation in vivo (Braley et al., 2016; LeBouder et al., 2003; Metz et al., 2012; Yang et al., 2017). A disintegrin and metalloprotease 10 (ADAM10) is the MMP suspected to be responsible for ectodomain shedding of PRRs at the cell surface, forming the solubilized isoforms of RAGE (cleaved RAGE; cRAGE) (Metz et al., 2012; Raucci et al., 2008). ADAM10 may also be involved with liberation of TLR2 (sTLR2) and TLR4 (sTLR4) along with posttranslational modification and alternative splicing mechanisms (Iwami et al., 2000; Langjahr et al., 2014). Alternative splicing of the RAGE transcript at exon 9 leads to the production of the stable endogenous secretory RAGE (esRAGE) isoform (Yonekura et al., 2003). esRAGE is truncated as it does not possess the c‐terminal transmembrane domain that is responsible for receptor integration into the membrane (Manigrasso et al., 2021; Ramasamy et al., 2016; Yonekura et al., 2003). Together, cRAGE and esRAGE comprise to the total soluble (sRAGE) population and once in the circulation are suspected to function similarly as sTLRs. Several studies have shown that aerobic exercise (AE) and stimulated muscle contractions augment circulating solubilized receptor concentrations (Choi et al., 2012; Ducharme et al., 2022; Kotani et al., 2011; Legaard et al., 2022; Santilli et al., 2013). While it is difficult to conclusively determine the origin of these solubilized receptors after appearing in the circulation, many tissues have been shown to contain or produce solubilized receptors (Henrick et al., 2016). Given skeletal muscle is a principle site of glucose disposal, comprises a significant portion of body mass, and is the predominant tissue facilitating movement and energy generation during exercise, as well as the exercise‐mediated hemodynamic changes, we suspect acute exercise initiates the machinery for the resolution of inflammation thereby leading to the ectodomain cleavage and release of these inflammatory membrane receptors into the circulating pool.

Several lines of evidence show solubilized receptor levels are inversely related to inflammatory burden and prevent metabolic dysfunction in isolated cells and animals (del Pozo et al., 2019; Iwami et al., 2000; Olekson et al., 2016; Song et al., 2014) and are inversely related to obesity, inflammatory conditions, and glucose intolerance in humans (Di Pino et al., 2016; Falcone et al., 2005; Miranda et al., 2017; Tan et al., 2006; ten Oever et al., 2014; Zaharieva et al., 2018). This effect is suspected to be driven by the antagonistic effects sRAGE and sTLRs exert on cellular signaling. More specifically, sRAGE and sTLRs may reduce membrane‐bound receptor signaling in four potential ways, by (1) reducing signaling at the cell surface due to fewer receptors, (2) acting as a decoy by binding and sequestering PAMPs/DAMPs in the circulation, (3) dimerizing with existing cell‐surface receptors—preventing receptors from binding with PAMPs/DAMPs, and/or (4) shedding of a PRR reduces dimerization opportunity of intact receptors needed to transduce signals intracellularly. Given the inflammatory nature of these receptors when membrane bound, and purported benefits of their solubilization, the purpose of this study was to explore the effect of an acute bout of AE on circulating sRAGE, sTLR2, and sTLR4 concentrations in normal‐ and abnormal‐glucose‐tolerant individuals. We hypothesized acute aerobic exercise would increase circulating sRAGE, sTLR2, and sTLR4 concentrations in both groups. Due to the limited amount of information on sRAGE and sTLRs, we also explored potential relationships among solubilized receptors and selected indices of metabolic health.

## 2 METHODS

### 2.1 Experimental design and participants

Demographic and clinical data from a subset of this studies participants have been previously reported (Eren‐Oruklu et al., 2009; Farabi et al., 2015; Moxley et al., 2018). Findings presented here represent a secondary analysis of this previously published work and is the first reporting of exercise responses related to this study's main outcomes. To determine eligibility, potential participants completed a detailed medical screening questionnaire and blood chemistry panel. Prior or current pulmonary, hepatic, renal, gastrointestinal, or hematologic disease, weight loss (>2 kg within 6 months), smoking, and contraindication to a strenuous exercise test were used as exclusion criteria. Once eligibility was confirmed, BMI‐matched participants were grouped based on glycemic control. Groups consisted of normal‐ (NGT, *n* = 10) and abnormal‐glucose‐tolerant (AGT, *n* = 16) individuals (Table 1). Abnormal glucose tolerance was used to include individuals classified as both impaired glucose tolerance and having type 2 diabetes according the American Diabetes Association classification and diagnosis of diabetes (ElSayed et al., 2023). All subjects provided written and oral informed consent to participate. The study was approved by the Institutional Review Board at the University of Illinois at Chicago and the University of Michigan and adhered to the principles outlined in the Helsinki Declaration.

**TABLE 1:** Subject characteristics.

	NGT (*n* = 10)	AGT (*n* = 16)
M/F	3/7	4/12
Age (years)	41 ± 4	51 ± 3*
Body mass (kg)	94.7 ± 5.0	95.0 ± 4.2
BMI (kg/m^2^)	35.6 ± 3.0	34.3 ± 1.5
Fat (%)	31.5 ± 1.9	39.9 ± 1.9*
VO_2_max (mL/kg/min)	28.1 ± 1.8	22.0 ± 1.6*
HbA1c (%)	5.2 ± 0.1	6.7 ± 0.3*
FPG (mg/dL)	92 ± 2	134 ± 9*
2 h OGTT (mg/dL)	116 ± 11	259 ± 18*
OGTT AUC (mg*h/dL)	249 ± 11	458 ± 29*
HOMA‐IR	2.94 ± 0.33	5.15 ± 0.74*
hsCRP (mg/L)	2.4 ± 0.6	2.0 ± 0.3

Abbreviations: AGT, abnormal glucose tolerant; BMI, body mass index; FPG, fasting plasma glucose; HOMA‐IR, homeostatic measure of insulin resistance; hsCRP, high‐sensitivity c‐reactive protein; Mean ± SE; NGT, normal glucose tolerant; OGTT, oral glucose tolerance test.

* *p* < 0.05 versus normal glucose tolerant.

### 2.2 Protocol overview and clinical testing procedures

**FIGURE 1:**
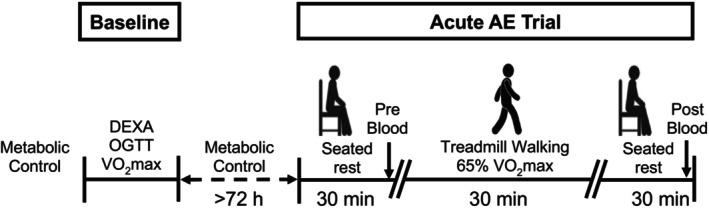
General study overview. Baseline testing consisted of body composition (DEXA), glucose metabolism (OGTT), and cardiovascular fitness (VO_2_max) assessment. Briefly, metabolic control preceded baseline testing and the acute aerobic exercise (AE) trial. This metabolic control period consisted of withholding oral antidiabetic drugs (24 h), refraining from consuming alcohol (>48 h) and caffeine (>24 h), and engaging in structured exercise (>24 h). The acute AE trial included 30 min treadmill walking at 65% VO_2_max. A pre‐ and post‐exercise blood draw was completed after 30 min of quiet, seated rest. See Section 2 for more details.

A general study overview is presented in Figure 1. Testing took place at the Clinical Research Unit at the University of Illinois at Chicago and the Michigan Clinical Research Unit at the University of Michigan. Participants being treated with oral antidiabetic drugs withheld their medications for >24 h prior to metabolic testing. In addition, all participants were instructed to refrain from consuming alcohol within 48 h, caffeine 24 h, and engaging in structured exercise for at least 24 h before metabolic testing.

Height and weight were assessed using standard techniques. Whole body adiposity was measured via dual‐energy X‐ray absorptiometry (Lunar iDXA; GE Healthcare, Madison, WI). Following an 8–10 h overnight fast, participants completed an oral glucose tolerance test (OGTT). Briefly, an antecubital venous catheter was inserted, and baseline blood was collected. Participants consumed 75 g anhydrous glucose dissolved in 300 mL water within ~5 min. Following glucose consumption, venous blood was sampled at regular intervals for 2 h (30‐, 60‐, and 120‐min post). Blood was centrifuged at 2000*g* for 15 min at room temperature. Following centrifugation, plasma/serum was stored at −80°C until analysis. Glucose AUC was determined for the OGTT using the trapezoidal calculation (Hulman et al., 2019; Sakaguchi et al., 2015). On a separate day, participants completed an incremental treadmill exercise test to determine maximal oxygen consumption (i.e., VO_2_max) as previously described (Solomon et al., 2015). VO_2_max testing was conducted at least 48 h prior to metabolically sensitive testing.

### 2.3 Acute aerobic exercise trial

Following baseline characterization of health and fitness, participants performed a supervised 30‐min treadmill exercise trial at 65% of VO_2_max on a separate visit (i.e., acute AE trial). This exercise bout was selected to provide a robust metabolic and cardiovascular challenge that aligns with American College of Sports Medicine and Physical Activity Guidelines for Americans (Kanaley et al., 2022; Piercy et al., 2018). Participants arrived having fasted 8–10 h, and a baseline blood draw was completed after 30 min quiet, seated rest. The treadmill exercise test immediately followed baseline blood collection, during which heartrate was monitored (Polar, Kempele, Finland) and confirmed with intermittent oxygen consumption assessment (Parvo Medics, Sandy, UT) to ensure the appropriate intensity was maintained. Adjustments to speed and/or incline were made as necessary throughout the test to achieve the target intensity. Following the acute AE trial, participants sat quietly for 30 min, after which a final blood draw was performed.

### 2.4 Blood collection and processing

Blood was collected from the antecubital vein into EDTA and Lithium coated vacutainers and SST tubes before and 30‐min after the acute AE trial. To account for blood volume shifting that occurs with changes in body position or engagement in exercise (Dill & Costill, 1974), participants were asked to sit quietly for 30 min prior to each draw. The 30‐min postexercise timepoint was selected to assess acute changes in circulating inflammatory factors while controlling for blood volume shifts (Dill & Costill, 1974) that occur with rapid changes in body position, including seat‐to‐standing and rest‐to‐exercise‐to‐rest. After collection, samples were centrifuged at 3000 rpm for 10 min at 4°C to isolate plasma, which was then stored at −80°C until further analysis.

### 2.5 Blood analyses

Circulating glucose was determined using a point‐of‐care (Count Next One, Bayer Healthcare, Mishawaka, IN) or bedside (YSI Stat; YSI, Yellow Springs, OH) analyzer. Insulin was assessed via enzyme‐linked immunosorbent assay (ELISA; #90095, Crystal Chem, Elk Grove Village, IL). hsCRP was determined via ELISA (#80955, Crystal Chem, Elk Grove Village, IL). Total plasma sRAGE was measured via ELISA (DRG00, R&D Systems, Minneapolis, MN, USA). This total sRAGE quantification approach captures cleaved (cRAGE) and endogenous secretory (esRAGE) isoforms. To quantify plasma esRAGE, another ELISA was performed (ABIN6971260, Antibodies Online, Limerick, PA). Plasma cRAGE was determined by subtracting esRAGE from total sRAGE (Fuller, Miranda, et al., 2018; Fuller, Valentine, et al., 2018; Miranda et al., 2017, 2019; Perkins et al., 2019). Plasma sTLR2 (ab131556, Abcam, Cambridge, MA) and plasma sTLR4 (MBS167606, My Biosource, San Deigo, CA) were also assessed via ELISA. sRAGE, cRAGE, esRAGE, sTLR2, and sTLR4 were all assessed in plasma collected in EDTA‐coated vacutainers. hsCRP was determined primarily (59%) in samples collected in LiHep (plasma) tubes, while the remainer of samples assessed were collected in EDTA (plasma; 20%) and SST (serum; 20%) due to sample availability.

### 2.6 Statistics

Data were analyzed with SPSS Statistics version 28.0 (IBM SPSS Inc, Armonk, NY). Subject characteristics were compared via independent *t*‐test. A mixed‐factorial two‐way analysis of variance (ANOVA) was performed. Data were checked for equal variance via Levene's test for equality. Pearson's correlations were used for correlative analysis. Linear regressions were used to determine the influence of independent variables of interest on outcome measures. Significance was set at *p* < 0.05, and a trend toward significance was recognized as *p* < 0.10. Data are presented as mean ± SE.

## 3 RESULTS

### 3.1 Subject characteristics

Subject characteristics are presented in Table 1. Though a subset of these data have been published previously, they are presented here for context to the study's main objective (Farabi et al., 2015). Participants were BMI‐matched (*p* > 0.05), but differed (% difference between groups, *p* < 0.05) with respect to age (AGT: +22%), body fat percentage (AGT: +24%), VO_2_max (AGT −24%), HbA1c percentage (AGT: +25%), OGTT AUC (AGT: +59%), 2 h OGTT (AGT: +76%), and HOMA‐IR (AGT: +55%). Medications used by AGT participants (*n* = 12) included metformin, glimepiride, dulaglutide, liraglutide, and empagliflozin.

### 3.2 Circulating inflammatory receptors

**FIGURE 2:**
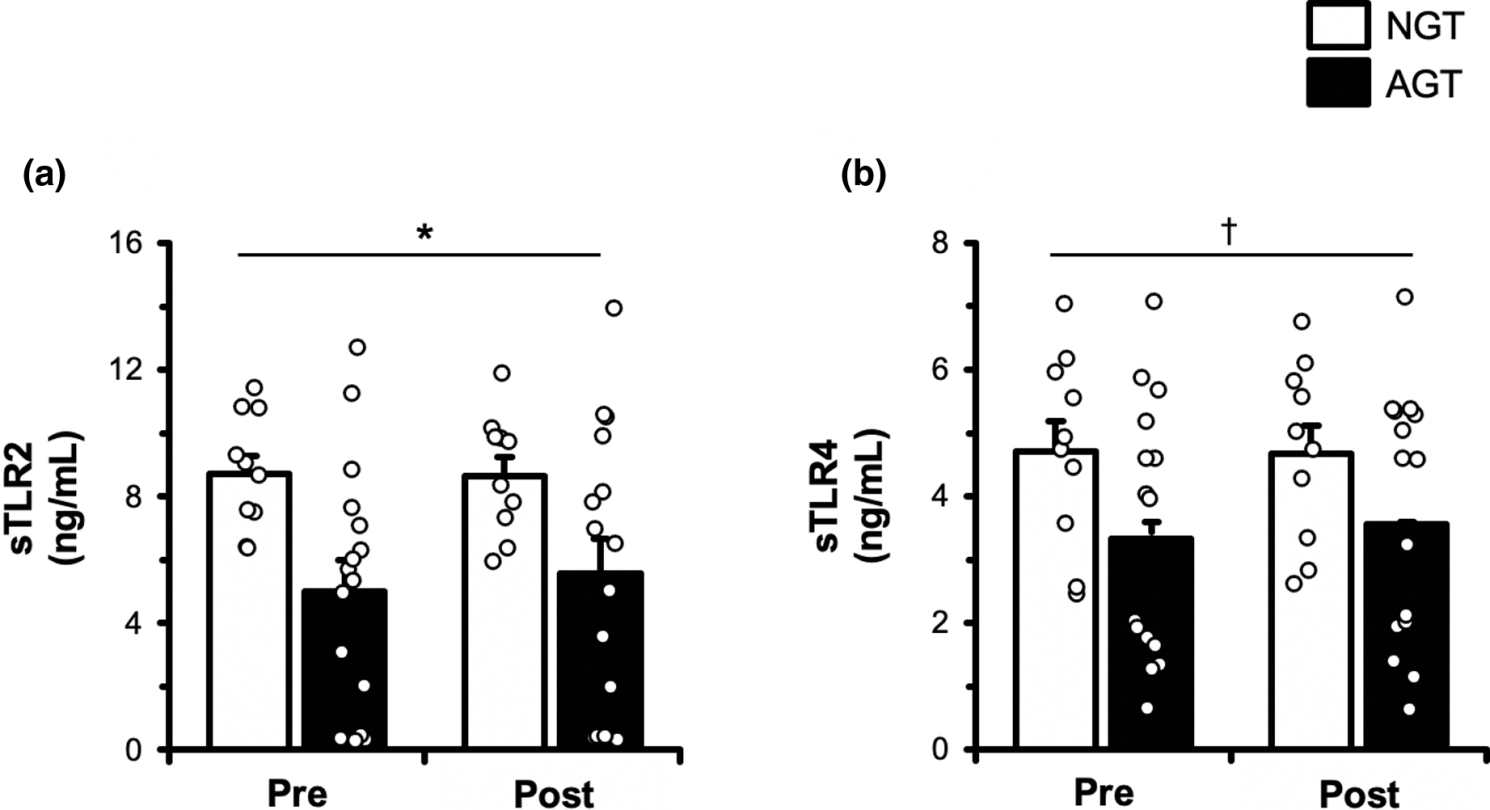
Plasma soluble Toll‐like receptor (sTLR)2 (a) and sTLR4 (b) in the basal state and 30 min postexercise in BMI‐matched normal‐glucose‐tolerant (NGT, *n* = 10) and abnormal‐glucose‐tolerant (AGT, *n* = 16) individuals. sTLR2 and sTLR4 were assessed via ELISA. See Section 2 for more details. Data are mean ± SE. *p < 0.05 main effect of group. ^†^ *p* < 0.10 trends toward main effect of group.

*sTLRs*: sTLR2 and sTLR4 data are presented in Figure 2. There was a main effect for group as sTLR2 was lower in AGT than NGT (*p* = 0.034; −33% overall). However, sTLR2 remained unchanged (*p* > 0.05) following exercise. There was a trend toward a main effect for group as sTLR4 tended to be lower in AGT than NGT (*p* = 0.096; −36% overall). However, sTLR4 remained unchanged (*p* > 0.05) following exercise.

### 3.3 sRAGE and isoforms

**FIGURE 3:**
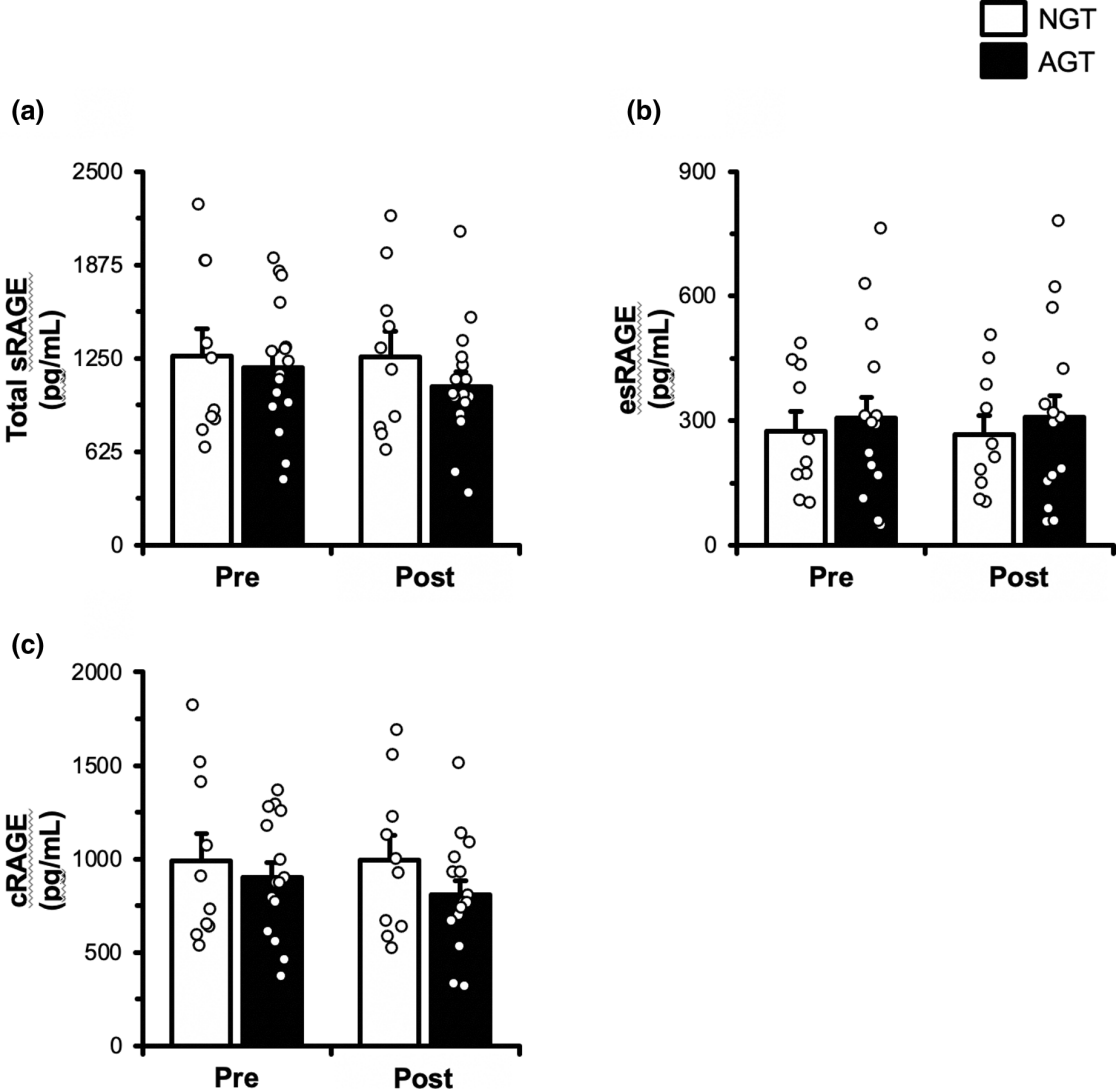
Total plasma soluble receptor for advanced glycation end products (sRAGE, a), endogenous secretory RAGE (esRAGE, b), and cleaved RAGE (cRAGE, c) in the basal state and 30 min postexercise in BMI‐matched normal‐glucose‐tolerant (NGT, *n* = 10) and abnormal‐glucose‐tolerant (AGT, *n* = 16) individuals. Total sRAGE and esRAGE were assessed via ELISA. cRAGE was calculated as the difference between total sRAGE and esRAGE. See Section 2 for more details. Data are mean ± SE.

Total sRAGE and isoforms are presented in Figure 3. Total sRAGE was similar (*p* > 0.05) between AGT and NGT at rest. Furthermore, total sRAGE remained unchanged in NGT but decreased a non‐significant 10% in AGT following exercise (*p* > 0.05). esRAGE was similar (*p* > 0.05) between AGT and NGT at rest and remained unchanged (*p* > 0.05) following exercise in both groups. cRAGE was similar (*p* > 0.05) between AGT and NGT at rest. cRAGE remained unchanged in NGT but decreased a non‐significant 9% in AGT following exercise (*p* > 0.05).

### 3.4 Select relationships among independent variables and primary outcomes

To explore potential relationships between independent variables of interest and primary outcome measures, we conducted correlation analyses (df = 24) with both groups combined (NGT + AGT) (Table 2). sTLR2 exhibited a strong relationship (*p* < 0.05) with age (*r* = −0.454), VO_2_max (*r* = 0.414), body fat % (*r* = −0.406), HbA1c (*r* = −0.510), basal blood glucose (*r* = −0.505), and OGTT AUC (*r* = −0.687) and did not appear to be related to HOMAIR (*r* = −0.116, *p* > 0.05). sTLR4 was related (*p* < 0.05) to age (*r* = −0.413), basal blood glucose (*p* < 0.10; *r* = −0.374), OGTT AUC (*r* = −0.402), and HDL (*r* = 0.453) and did not appear to be related to HOMAIR (*r* = 0.247, *p* > 0.05). Total sRAGE and sRAGE isoforms did not exhibit any apparent meaningful relationships with subject characteristics (*p* > 0.05). Next, we performed multiple linear regression analysis to determine whether independent variables of interest (age, body fat %, HbA1c, OGTT AUC, HOMAIR, basal BG, relative VO_2_max, and HDL) were predictive of sTLR2 and sTLR4 (inflammatory factors that exhibited a main effect) (Table S1 and S2). Independent variables were found to predict sTLRs in the basal state (sTLR2: *F*(8, 17) = 3.800, *p* = 0.01; sTLR4: *F*(8, 17) = 2.769, *p* = 0.037) and after acute exercise state (sTLR2: *F*(8, 17) = 4.536, *p* = 0.004; sTLR4: *F*(8, 17) = 2.789, *p* = 0.036). Moreover, the *R* ^2^ of basal sTLR2 (0.641) and sTLR4 (0.566) and postexercise sTLR2 (0.681) and sTLR4 (0.568) depicts that the models explain ~57%–68% of the variation in sTLR2 and sTLR4 levels.

**TABLE 2:** Select relationships between solubilized receptors and characteristics of interest.

	sTLR2	sTLR4	Total sRAGE	esRAGE	cRAGE
Age (years)	−0.454*	−0.413*	−0.040	0.177	−0.045
VO_2_max (mL/kg/min)	0.414*	0.068	0.080	0.061	0.083
Body fat (%)	−0.406*	−0.180	−0.240	−0.123	−0.248
HbA1c (%)	−0.510*	−0.308	0.096	0.152	0.022
Basal BG (mg/dL)	−0.505*	−0.374^†^	0.068	0.162	0.111
OGTT AUC (mg*h/dL)	−0.687*	−0.402*	0.007	0.156	−0.027
HDL (mg/dL)	0.277	0.453*	−0.105	0.013	−0.102

*Note*: Pearson correlation coefficient; *p < 0.05; ^†^ *p* < 0.10.

## 4 DISCUSSION

Perpetuation of inflammatory activities via RAGE and TLR signaling are suspected to contribute to the development and progression of metabolic disease, including diabetes. Exercise has been shown to augment circulating solubilized receptor concentrations (Choi et al., 2012; Ducharme et al., 2022; Kotani et al., 2011; Legaard et al., 2022; Santilli et al., 2013), thereby reducing membrane‐bound signaling. The primary goal of this investigation was to explore the effects of an acute bout of aerobic exercise on circulating sRAGE, sTLR2, and sTLR4. Interestingly, we show that sTLR2 and sTLR4 are lower in abnormal‐glucose‐tolerant than normal‐glucose‐tolerant individuals. Furthermore, sTLR2 and sTLR4 were strongly related to multiple indices of metabolic and cardiovascular health.

Tissue‐bound RAGE and TLRs are tasked with modulating the innate immune response, and therefore, their activity has substantial metabolic implications. Dysregulated RAGE and TLR signaling are suspected to perturb the inflammatory environment classically seen in those with insulin resistance, thereby contributing to the development and progression of insulin resistance (Cassese et al., 2008; Creely et al., 2007; Giacco & Brownlee, 2010; Jialal et al., 2014; Schmidt et al., 2001). Therefore, shedding RAGE and TLRs from the cell surface or producing a solubilized isoform via alternative splicing or posttranslational modification has an overall anti‐inflammatory effect. It is important to note that many tissues have been shown to contain or produce solubilized receptors. Given skeletal muscle is a principle site of glucose disposal, comprises a significant portion of body mass, and is the predominant tissue facilitating movement and energy generation during exercise, we suspect acute exercise activates MMP and ADAM signaling in muscle, thereby leading to the release of these membrane receptors into the circulating pool (Henrick et al., 2016). Here, we show that circulating sTLR2 and sTLR4 are 35%–54% lower in individuals with apparent abnormal glucose regulation than otherwise healthy obese individuals. These findings are in line with our previous work on sRAGE (Miranda et al., 2017), showing that development/progression along the glucose tolerance continuum leads to reduced circulating PRR levels. Furthermore, to our knowledge only one other report exists on sTLRs and insulin resistance. In agreement with findings presented here, this report shows that sTLR2 is ~22% lower in T2DM than age‐matched control participants. However, unlike findings presented here, there was no difference in sTLR4 between the T2DM and control groups (Zaharieva et al., 2018). Potential explanations for the discrepant findings between those reported here and the Zaharieva et al. study include a higher BMI in our participants and withholding of medications (i.e., metabolic control) before our assessment of circulating factors. Though an incomplete understanding exists, data exist showing that TLR shedding is modulated by pharmacological drugs (Langjahr et al., 2014), which highlights the importance in controlling for factors that influence production of solubilized receptors.

To our knowledge, this is the first report on circulating sTLR and sRAGE levels in individuals with apparent abnormal glucose regulation following acute aerobic exercise. However, we recently demonstrated that while young individuals with obesity have higher muscle RAGE protein expression, they do not appear to exhibit different circulating sRAGE levels (Miranda et al., 2023). Furthermore, muscle RAGE expression was strongly related to HOMA‐IR, in disagreement with findings presented here. This is thought‐provoking and suggests altered solubilized PRR levels may not be observable until disease/inflammatory states have progressed (i.e., abnormal glucose tolerant and T2DM). Interestingly, a modest bout of acute AE reduced circulating sRAGE levels at lower exercise intensities, unlike findings presented here. Given the findings from Miranda et al and those presented here show no change in sRAGE and/or sTLRs following AE, it remains unclear as to how aerobic exercise training effectively increases basal circulating sRAGE levels (Choi et al., 2012; Legaard et al., 2022). ADAM10 has been reported to responsible for proteolytic cleavage of RAGE from the extracellular domain of tissue (Metz et al., 2012; Raucci et al., 2008) via calcium calmodulin kinase (CAMK) and G‐protein coupled receptor (GPCR) mechanisms. CAMK and GPCR signaling is upregulated in skeletal muscle during AE due to the classic hormonal response (i.e., antidiuretic hormone and epinephrine) and given muscles involvement in exercise, is suspected to be an appreciable source of solubilized receptors. When activated in vivo, cells cleave RAGE from the cell surface, increasing appearance of cRAGE in the cell media. Given this preclinical data, it was surprising that sRAGE did not change following exercise. Potential explanations for this finding include myokine trapping in the interstitial space once cleaved from the membrane, time course dependent shedding mechanisms not captured within our study design, vascular dysfunction in those with insulin resistance (Padilla et al., 2022), and/or augmented renal sRAGE clearance (Kalousova et al., 2006; Perkins et al., 2019, 2023). While we controlled for acute plasma volume shifts, future work is encouraged to expand the timeframe following exercise.

In general, sTLR2 and sTLR4 correlated strongly with multiple indices of metabolic and cardiorespiratory health. Interestingly, sTLR2 has been found to be lower in heart failure patients with a recent myocardial infarction than healthy controls (Ueland et al., 2006). Taken together, these data suggest sTLR2 may be a good circulating marker for cardiometabolic health, warranting future research. sTLR2 and sTLR4 exhibited a clear relationship with factors related to glycemic control. More specifically, sTLR2 was inversely related to HbA1c, basal blood glucose, and the OGTT AUC, whereas sTLR4 was also inversely related to basal blood glucose and the OGTT AUC. Multiple linear regression analyses showed that indices of metabolic health (i.e., OGTT AUC, HOMAIR, and basal blood glucose) significantly contributed to our predictive model of sTLR2 and sTLR4. Given the evidence that TLR4 activation suppresses insulin action in myotubes (Hussey et al., 2012), findings presented here provide context for the potential preservation of insulin sensitivity by disrupting PRR signaling via solubilization strategies.

While these data contribute to the limited amount of work on sRAGE and sTLRS with exercise and insulin resistance, it is important to note that this study is not without limitations. First, we selected a modest bout of exercise (i.e., 30 min AE at 65% VO_2_max) that aligns with standard physical activity guidelines (Kanaley et al., 2022; Piercy et al., 2018). While this type of exercise stimulus leads to favorable cardiovascular and tissue metabolic adaptations when completed regularly, it may not exert a robust enough inflammatory response to activate ADAM10, alternative splicing, and/or post‐transcriptional events that upregulate sRAGE and sTLR production. Next, we cannot state conclusively the source of circulating sRAGE and sTLRs. Though skeletal muscle is a predominant tissue by mass and metabolic activity in humans and these solubilized receptors have been reported to be muscle‐derived, other tissues (i.e., adipose, vascular, and immune cells) may also contribute to the circulating sRAGE and sTLR pool. Our groups also differed in age by 10 years, and our AGT clustering included individuals with type 2 diabetes. While age was not a significant contributor to our linear regression analyses of sTLR2 and sTLR4, it is possible this age difference exerted an effect. Also, we cannot discount the potential influence of antidiabetic medications on soluble receptor biology. Lastly, though we controlled for plasma volume shifting typically observed with exercise, it is possible our postexercise assessment period missed appearance/accumulation of sRAGE and sTLRs into the circulation.

In summary, we sought to characterize the circulating sRAGE and sTLR profile in normal and abnormal‐glucose‐tolerant individuals in the basal state and after a standard bout of AE. We show that circulating sTLR2 and sTLR4 are lower in abnormal‐glucose‐tolerant participants, a modest bout of acute AE does not change sTLR and sRAGE levels, and these solubilized receptors are strongly related to indices of health. Our findings shed light on the circulating inflammatory environment in those with altered glucose disposal/metabolism and given their role in maintaining metabolic homeostasis, may contribute to development and/or progression of diseases related to glucose intolerance. Future work is encouraged to incorporate a wider range of AE challenges (i.e., intensity and duration), extend the timeframe after AE to assess the potential changes in circulating sRAGE and sTLR levels, and evaluate the role the acute response plays in adaptation.

## AUTHOR CONTRIBUTIONS

*Experimental design*: Ryan K. Perkins, Edwin R. Miranda, Lauretta T. Quinn, Jacob M. Haus. *Data collection*: Ryan K. Perkins, Edwin R. Miranda, Pallavi Varshney, Sarah S. Farabi, Lauretta T. Quinn, Jacob M. Haus. *Analyzed data*: Ryan K. Perkins, Edwin R. Miranda, Pallavi Varshney, SF. *Manuscript preparation*: Ryan K. Perkins, Jacob M. Haus. *Funding acquisition*: Lauretta T. Quinn, Jacob M. Haus. All authors have read and agreed to the final version of the manuscript.

## FUNDING INFORMATION

R01 DK109948, R01 NR07760.

## CONFLICT OF INTEREST STATEMENT

The authors have no conflicts of interest to declare.

## Supporting information


Tables S1‐S2.

## Data Availability

Data will be made available by authors upon reasonable request.
