# Supplementary figures and images for: Effects of acute aerobic exercise on circulating sTLR and sRAGE profiles in normal‐ and abnormal‐glucose‐tolerant individuals
Source: Physiol Rep. 2023 Nov 20;11(22):e15859. doi: 10.14814/phy2.15859 (PMC10659941; doi:10.14814/phy2.15859)

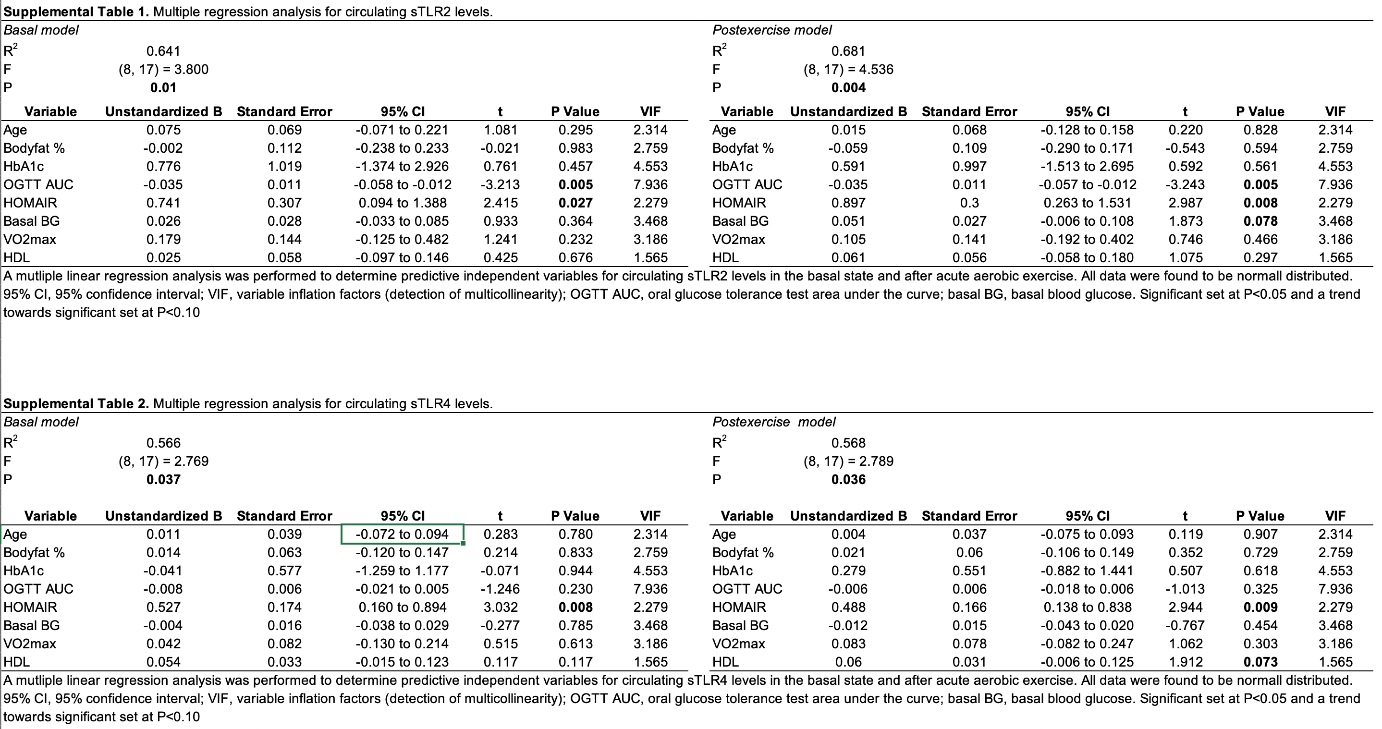


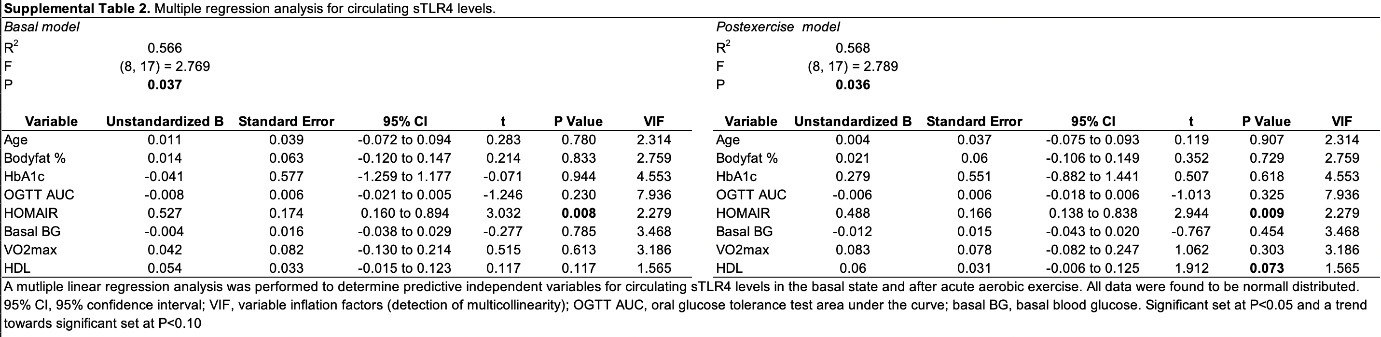

Supplement: Supplementary file 1 — Tables S1‐S2. [file PHY2-11-e15859-s001.docx]
